# Plant–soil feedback regulates the trade-off between phosphorus acquisition pathways in *Pinus elliottii*

**DOI:** 10.1093/treephys/tpad044

**Published:** 2023-04-19

**Authors:** Ning Ma, Liang Kou, Shenggong Li, Xiaoqin Dai, Shengwang Meng, Lei Jiang, Yafang Xue, Jiajia Zheng, Xiaoli Fu, Huimin Wang

**Affiliations:** National Ecosystem Science Data Center, Key Laboratory of Ecosystem Network Observation and Modeling, Institute of Geographic Sciences and Natural Resources Research, Chinese Academy of Sciences, Beijing 100101, China; College of Resources and Environment, University of Chinese Academy of Sciences, Beijing 100049, China; Sino-Danish Center for Education and Research, Eastern Yanqihu Campus, University of Chinese Academy of Sciences, 380 Huaibeizhuang, Beijing 101400, China; College of Resources and Environment, University of Chinese Academy of Sciences, Beijing 100049, China; Qianyanzhou Ecological Research Station, Key Laboratory of Ecosystem Network Observation and Modeling, Institute of Geographic Sciences and Natural Resources Research, Chinese Academy of Sciences, Beijing 100101, China; National Ecosystem Science Data Center, Key Laboratory of Ecosystem Network Observation and Modeling, Institute of Geographic Sciences and Natural Resources Research, Chinese Academy of Sciences, Beijing 100101, China; College of Resources and Environment, University of Chinese Academy of Sciences, Beijing 100049, China; Sino-Danish Center for Education and Research, Eastern Yanqihu Campus, University of Chinese Academy of Sciences, 380 Huaibeizhuang, Beijing 101400, China; Qianyanzhou Ecological Research Station, Key Laboratory of Ecosystem Network Observation and Modeling, Institute of Geographic Sciences and Natural Resources Research, Chinese Academy of Sciences, Beijing 100101, China; Qianyanzhou Ecological Research Station, Key Laboratory of Ecosystem Network Observation and Modeling, Institute of Geographic Sciences and Natural Resources Research, Chinese Academy of Sciences, Beijing 100101, China; National Ecosystem Science Data Center, Key Laboratory of Ecosystem Network Observation and Modeling, Institute of Geographic Sciences and Natural Resources Research, Chinese Academy of Sciences, Beijing 100101, China; National Ecosystem Science Data Center, Key Laboratory of Ecosystem Network Observation and Modeling, Institute of Geographic Sciences and Natural Resources Research, Chinese Academy of Sciences, Beijing 100101, China; College of Resources and Environment, University of Chinese Academy of Sciences, Beijing 100049, China; National Ecosystem Science Data Center, Key Laboratory of Ecosystem Network Observation and Modeling, Institute of Geographic Sciences and Natural Resources Research, Chinese Academy of Sciences, Beijing 100101, China; College of Resources and Environment, University of Chinese Academy of Sciences, Beijing 100049, China; College of Resources and Environment, University of Chinese Academy of Sciences, Beijing 100049, China; Qianyanzhou Ecological Research Station, Key Laboratory of Ecosystem Network Observation and Modeling, Institute of Geographic Sciences and Natural Resources Research, Chinese Academy of Sciences, Beijing 100101, China; College of Resources and Environment, University of Chinese Academy of Sciences, Beijing 100049, China; Qianyanzhou Ecological Research Station, Key Laboratory of Ecosystem Network Observation and Modeling, Institute of Geographic Sciences and Natural Resources Research, Chinese Academy of Sciences, Beijing 100101, China

**Keywords:** ectomycorrhizal fungi, pathogenic fungi, phosphorus acquisition strategy, phosphorus addition, plant–soil feedback, soil fungal community

## Abstract

Plant–soil feedback (PSF) is conventionally characterized by plant biomass growth, yet it remains unclear how PSF affects plant nutrient acquisition strategies (e.g., nutrient absorption and nutrient resorption) associated with plant growth, particularly under changing soil environments. A greenhouse experiment was performed with seedlings of *Pinus elliottii* Englem and conditioned soils of monoculture plantations (*P. elliottii* and *Cunninghamia lanceolata* Hook). Soil sterilization was designed to test plant phosphorus (P) acquisition strategy with and without native soil fungal communities. Soils from *P. elliottii* and *C. lanceolata* plantations were used to explore the specific soil legacy effects on two different P acquisition pathways (absorption and resorption). Phosphorus addition was also applied to examine the separate and combined effects of soil abiotic factors and soil fungal factors on P acquisition pathways. Due to diminished mycorrhizal symbiosis, PSF prompted plants to increasingly rely on P resorption under soil sterilization. In contrast, P absorption was employed preferentially in the heterospecific soil, where species-specific pathogenic fungi could not affect P absorption. Higher soil P availability diluted the effects of soil fungal factors on the trade-off between the two P acquisition pathways in terms of the absolute PSF. Moreover, P addition plays a limited role in terms of the relative PSF and does not affect the direction and strength of relative PSF. Our results reveal the role of PSF in regulating plant P acquisition pathways and highlight the interaction between mycorrhizal and pathogenic fungi as the underlying mechanism of PSF.

## Introduction

In terrestrial ecosystems, plants can alter the composition of the native soil microbial community, which in turn affects plant growth and leads to plant–soil feedbacks (PSFs) (Bever 2003, Kulmatiski et al. 2008, van der Putten et al. 2013). The direction and strength of PSF are driven mainly by two fungal functional groups, namely pathogenic and mutualistic fungi (Heinen et al. 2020). Pathogenic fungi often cause negative feedback, whereas mutualistic fungi such as mycorrhizal fungi mostly result in positive feedback, and this may potentially neutralize the negative feedback induced by other pathogenic fungi (Bennett and Klironomos 2019, Thakur et al. 2021). However, empirical studies on PSF focus mostly on plant functions such as biomass growth and less on nutrient-associated processes such as nutrient absorption and nutrient resorption whose interactions can influence plant growth (Pernilla Brinkman et al. 2010, van der Putten 2017, Kandlikar et al. 2019). These two processes represent two major nutrient acquisition pathways of plants, and their trade-off may depend on complex interactions between plants and soil environment (Kou et al. 2017). Despite this, our current knowledge regarding the trade-off between the two pathways under the PSF framework remains limited.

Absolute PSF influences plant nutrient acquisition strategies through the net outcome of mutualistic and pathogenic fungi in the conspecific soil (Semchenko et al. 2018, Bennett and Klironomos 2019, Crawford et al. 2019). Mutualistic fungi, especially mycorrhizal fungi, contribute to nutrient absorption via the expansion of soil contact area by hyphae and the transformation of nutrients from organic to inorganic forms, leading to a positive absolute PSF (Laliberté et al. 2015, Lindahl and Tunlid 2015, Jiang et al. 2021). However, pathogenic fungi exert detrimental effects on nutrient absorption by inducing functional loss of roots and decreasing mycorrhizal symbiosis, resulting in a negative absolute PSF (Raaijmakers et al. 2009). In addition, pathogenic and mycorrhizal fungi may compete for carbon and infection sites on host plants (van der Putten et al. 2016, Teste et al. 2017). In this case, the trade-off between plant nutrient acquisition pathways in unsterilized soil depends on the outcome of the competition between mycorrhizal and pathogenic fungi (Semchenko et al. 2018, Chen et al. 2019). Dominant mycorrhizal fungi in the fungal community encourage nutrient absorption (Hodge and Storer 2015). However, the prevailing pathogenic fungi have the opposite effect on nutrient absorption, which indirectly enhances nutrient resorption (Cao et al. 2015). Nutrient addition might also induce the proliferation of pathogenic fungi (Balzergue et al. 2013, Zheng et al. 2017, Mujica et al. 2020). Surrounded by the native soil fungal community, plants grown in the unsterilized conspecific soil inhibit the establishment and persistence of mycorrhizal fungi and reject hosting them under fertilization.

Nevertheless, without the interference of pathogenic and mycorrhizal fungi, plants grown in sterilized soil will absorb nutrients unassisted. In such cases, the trade-off between the two nutrient acquisition pathways may depend on carbon cost and edaphic nutrient conditions (Wright and Westoby 2003, Kou et al. 2017). Plants may invest more in nutrient absorption by roots but less in nutrient resorption from leaves. This accelerates the rate of nutrient acquisition and reduces carbon expenditure in nutrient-deficient soils. By contrast, this trade-off may not take place in nutrient-rich soil because high nutrient availability forces plants to increase reliability on nutrient absorption through the roots. This reduces nutrient resorption in the sterilized conspecific soil (Smith-Ramesh and Reynolds 2017).

With the exception of the native soil fungal community, different plant species leave specific soil legacies in the fungal community that significantly influence nutrient acquisition strategies. The species-specific soil fungal community gives rise to the relative PSF. It can be verified by comparing the soil fungal communities from the same plant species when it is grown both in unsterilized (live) conspecific and heterospecific soils (Bennett and Klironomos 2019, Heinen et al. 2020). In addition to abundant mycorrhizal fungi, a large number of host-specific pathogenic fungi accumulate in unsterilized conspecific soils in the context of long-term monocultures (Semchenko et al. 2018). It is often identified as the primary cause of negative relative PSF (Aldorfová and Münzbergová 2019, Kandlikar et al. 2019, van der Putten et al. 2013). Plant nutrient resorption in the conspecific soil often help save carbon captured by accumulative pathogenic fungi. In addition, high soil fertility creates a favorable condition for the proliferation of pathogenic fungi that impairs nutrient absorption but facilitates nutrient resorption (Veresoglou et al. 2013). Even with few pathogenic fungi, symbiotic associations are diminished in the heterospecific soil. This is due to the limited function by mycorrhizal fungi in the unfamiliar and hyperdiverse soil fungal community in a new range, contributing to the positive relative PSF (Kadowaki et al. 2018). Alternatively, if plants acclimatize to the heterospecific soil and attempt to recruit other mutualistic fungi, nutrient absorption will occur. Nutrient addition might not trigger any variation in interactions between the heterospecific soil fungal community and the host plant because mycorrhizal and pathogenic fungi are less responsive to the exotic plant species (Semchenko et al. 2018). Therefore, plants in unsterilized heterospecific soil under fertilization adopt nutrient acquisition pathways equally.

Previous studies suggested that herbs suffered more from negative PSFs compared with trees (Kulmatiski et al. 2008). This might result from their arbuscular mycorrhizal (AM) associations (Bennett et al. 2017). Therefore, we chose *Pinus elliottii* Englem, an ectomycorrhizal (ECM) tree, which has a higher capacity than AM trees to exploit organic phosphorus (P) and has been the representative ECM species of local plantation for vegetation restoration since 1985 (Liu et al. 2018, Yan et al. 2022, Jiang et al. 2023). We examined P acquisition in an ECM tree and provided an illustration for PSF study. In the past decades, P limitation has strongly restricted the growth of plants in subtropical forests (Li et al. 2016*a*, Du et al. 2020). Humid climate in conjunction with nitrogen deposition aggravates the situation (Vitousek et al. 2010, Zhu et al. 2015). We conducted a greenhouse experiment in a subtropical plantation ecosystem to explore the role of PSFs in nutrient acquisition pathways of plants. Seedlings of *P. elliottii* were grown in the sterilized conspecific soil and the unsterilized conspecific soil from underneath *P. elliottii* trees (ECM), as well as unsterilized heterospecific soil underneath *Cunninghamia lanceolata* Hook trees (AM). We attempted to examine the effects of absolute PSFs (with vs without native soil microbiota) and relative PSFs (‘home’ vs ‘foreign’ soil microbiota) on two nutrient acquisition pathways. Phosphorus addition was also further manipulated to investigate the effects of soil nutrient availability on nutrient acquisition in terms of PSFs. The direction and strength of PSFs were assessed based on the root–soil accumulation factor (RSAF), which is indicative of nutrient absorption and nutrient resorption efficiency (NuRE). The effect of soil fungal factors (SFFs) on PSFs regarding the relative abundance of ECM fungi and pathogenic fungi was also evaluated. We hypothesized that: (i) absolute PSF on nutrient absorption would be positive, and that on nutrient resorption would be negative, with regards to the increased nutrient absorption and the decreased nutrient resorption influenced by native soil microorganisms; (ii) relative PSF on nutrient absorption would be negative, and that on nutrient resorption would be positive, indicating that plants preferentially employ nutrient absorption rather than nutrient resorption with the assistance of heterospecific soil microbiota; and (iii) P addition would transform absolute PSF to the opposite direction but not the strength. However, P addition would only affect the strength of relative PSF, driving plants grown in the live conspecific soil to be more inclined toward nutrient resorption than nutrient absorption.

## Materials and methods

### Study site description

The experiment was conducted in a greenhouse of Qianyanzhou Subtropical Forest Ecosystem Observation and Research (Qianyanzhou Station), Chinese Academy of Sciences, Taihe County, Jiangxi province of southeastern China (26°44′29.1′′N, 115°03′29.2′′E). The site has a typical subtropical moist monsoon climate with a mean annual air temperature of 17.9 °C and a mean annual precipitation of 1475 mm. The soil in the area is classified as Typical Hapludult Ultisol, weathered from red sandstone (Wang et al. 2012). Humid climate and long-term nitrogen deposition further exacerbate P limitation in the highly weathered red soil (Kou et al. 2015, Wang et al. 2019). The original vegetation is evergreen broad-leaved forests. However, these forests have been significantly reduced due to logging and reclamation. *Pinus elliottii* Englem (ECM tree species), *Pinus massoniana* Lamb (ECM tree species) and *C. lanceolata* Hook (AM tree species) have been the main afforestation species used for vegetation restoration since 1985 (Jiang et al. 2018, Zheng et al. 2022).

### Experimental design and treatment

Phosphorus acquisition was our main research object. Topsoil was collected from *C. lanceolata* and *P. elliottii* plantations using shovels at a depth of 0–10 cm from around adult trees. After collection, half of the soil from the *P. elliottii* plantations was sterilized using wet autoclaving at 120 °C for 1 h and 100 °C for 1 h. The soil was then mixed with sand in a 2:8 (soil:sand) ratio before the experiment starts to ensure that preconditions were equal for all pots. The pots had an external diameter of 20 cm and a height of 14 cm, with each pot filled with 2 kg mixture of soil and sand. Primary data of soil mixture physicochemical properties are shown in Table S1 available as Supplementary data at *Tree Physiology* Online. In May of 2019, healthy 1-year seedlings of *P. elliottii* (10–20 cm in height and 5–10 g in fresh weight) grown in a nursery near the Qianyanzhou Station with the same ambient conditions were then selected for the experiment. Before being transplanted into pots, their roots were sterilized in 1% sodium hypochlorite and rinsed with water afterwards (Bezemer et al. 2018). Each combination had four replicates, with one seedling in each pot. Therefore, a completely randomized design with six combinations was used in this study, including one plant species (*P. elliottii* saplings) × three soil treatments (the unsterilized soils from *C. lanceolata* and *P. elliottii* plantations, and the sterilized soil from *P. elliottii* plantation) × two P addition levels (control and addition of 2.3 mg P ml^−1^ referring to the P concentration demanded in Hoagland’s solution). Phosphorus was added for the relevant treatments every 2 months.

Litterfall of *P. elliottii* was collected once at appearance and then pooled for each individual separately. After 15 months of the feedback phase, we harvested the saplings in October 2020. Fine roots (<2 mm) and leaves are separately collected. All plant samples were dried at 65 °C for 48 h. Soil samples taken from each pot were divided into two parts. One part was dried for chemical analysis, and the other was stored at −20 °C for DNA extraction.

### Calculation of P absorption, resorption and PSFs

Dried plant and soil samples were ground into a fine powder using a Retsch MM400 mixer mill (Retsch GmbH, Haan, Germany). The concentration of total P was determined via inductively coupled plasma-optical emission spectrometry (Perkin Elmer Optima 5300 DV) after microwave acid-digestion of the samples in concentrated HNO_3_ and HClO_4_.

The RSAF was calculated to explore the difference between the P stored in the soil and the P absorbed by fine roots, which indicates the P absorption (Kou et al. 2017, Jiang et al. 2023). The RSAF is modified from the bioconcentration factor that is often used in the calculation of pollutants (Chen et al. 2015, Eid et al. 2021, Rasool et al. 2021). The RSAF was calculated as ‘${P}_{FR}/{P}_{soil}$’, where ${P}_{FR}$ and ${P}_{soil}$ were respectively the P concentration in fine roots and in the soil.

The nutrient conservation pathway can be described by NuRE (Wang et al. 2019), which is the percentage of nutrients withdrawn from senescent leaves relative to green leaves, including phosphorus resorption efficiency (*PRE*) (Aerts and Chapin 2000).


(1) \begin{equation*} PRE=\frac{\left({P}_G-{P}_{sen}\right)\times MLCF}{P_G}\times 100 \end{equation*}

where ${P}_G\ \mathrm{and}\ {P}_{sen}$ are the concentration of P in green leaves and senescent leaves, respectively. A mass loss correction factor (*MLCF*) is used to compensate for the underestimation of NuRE because of the reduction of leaf mass during senescence. The MLCF of conifers was found to be 0.65 (Zhou et al. 2021).

Plant–soil feedback values were calculated from each of four replicates in unsterilized conspecific soil versus the average of four replicates in sterilized conspecific or unsterilized heterospecific soil for each seedling (Png et al. 2018). Absolute PSF (${\mathrm{PSF}}_{\mathrm{absolute}}$) and relative PSF (${\mathrm{PSF}}_{\mathrm{relative}}$) were calculated in terms of RSAF (hereinafter referred to as ‘PSF on absorption’) or PRE (hereinafter referred to as ‘PSF on resorption’) as:


(2) \begin{equation*} {PSF}_{absolute}=\mathit{\ln}\frac{I_{c(u)}}{\overline{I_{c(s)}}} \end{equation*}

(3) \begin{equation*} {PSF}_{relative}=\mathit{\ln}\frac{I_c}{\overline{I_h}} \end{equation*}

where ${I}_{\mathrm{c}\left(\mathrm{u}\right)}$ and $\overline{I_{\mathrm{c}\left(\mathrm{s}\right)}}$ refer to indicator (RSAF or PRE) values of each individual *P. elliottii* in the unsterilized conspecific (*P. elliottii*) soil and the average indicator value of four *P. elliottii* in the sterilized conspecific soil, respectively. ${I}_{\mathrm{c}}$ and $\overline{I_{\mathrm{h}}}$ refer to indicator (RSAF or PRE) values of each individual *P. elliottii* in the conspecific soil and the average indicator value of four *P. elliottii *in the heterospecific (*C. lanceolata*) soil. Positive feedback values mean that RSAF or PRE in the unsterilized and conspecific soil was higher than in the sterilized conspecific or unsterilized heterospecific soil. Negative feedback values represent the lower RSAF or PRE in the unsterilized and conspecific soil than in the sterilized conspecific or unsterilized heterospecific soil. Opposite directions of absolute PSFs on absorption and resorption indicate the trade-off between two P acquisition pathways under the regulation of PSF.

### Fungal identification

Total DNA content was extracted by using a Fast DNA® SPIN kit (MP Biomedicals, USA), according to the manufacturer’s instructions. The concentration and purity of extracted DNA were determined using NanoDrop 2000 UV–vis spectrophotometer (Thermo Scientific, Wilmington, DE, USA). Primer pairs ITS1F (CTTGGTCATTTAGAGGAAGTAA) and ITS2R (GCTGCGTTCTTCATCGATGC) were used for polymerase chain reaction (PCR) amplification to determine the abundance and composition of the soil fungal community. PCR tests were performed in triplicate. The PCR comprised 20 μl mixtures containing 2 μl of 10 × Buffer (ITS), 2 μl of dNTPs (2.5 mM), 0.8 μl each of forwarding primer and reverse primer (5 μM), 0.2 μl of rTaq Polymerase (ITS), 0.2 μl of BSA, 10 ng Template DNA. Furthermore, each sample was supplemented with up to 20 μl of ddH_2_O. The PCR amplifications were conducted using a specific thermal process, which comprised: denaturation at 95 °C for 3 min, followed by 35 cycles of deformation at 95 °C for 30 s, annealing at 55 °C for 30 s, elongation at 72 °C for 45 s and a final extension at 72 °C for 10 min. The product was mixed and detected using a 2% agarose gel electrophoresis and was then purified using an AxyPrep DNA Gel Extraction kit (Axygen Biosciences, Union City, CA, USA). This analysis was undertaken according to the manufacturer’s instructions and quantified using a Quantus™ Fluorometer (Promega, USA). Purified amplicons were pooled, and equimolar and paired-end sequencing was done using an Illumina MiSeq PE300 platform (Illumina, San Diego, CA, USA) according to the standard protocols by Majorbio Bio-Pharm Technology Co. Ltd (Shanghai, China).

Functional groups of fungal operational taxonomic units (OTUs) were categorized into three trophic modes: pathotrophs, saprotrophs and symbiotrophs. These were divided into 12 guilds, including AM and ECM, based on the FUNGuild database (Nguyen et al. 2016). The OTUs were categorized according to the confidence levels of highly probable, probable and possible and those without confidence levels were removed from the analyses. Within these constraints, FUNGuild was found to be able to infer 59.4% of the OTUs (865/1456).

Absolute SFF and relative SFF were used to measure the impacts of SFFs on PSFs. It was done by imitating the calculation of PSFs. Absolute SFF (SFF_absolute_) and relative SFF (SFF_relative_) were respectively calculated as:


(4) \begin{equation*} {SFF}_{absolute}=\mathit{\ln}\frac{I_{P(u)}}{\overline{I_{P(s)}}} \end{equation*}

(5) \begin{equation*} {SFF}_{relative}=\mathit{\ln}\frac{I_P}{\overline{I_{Cun}}} \end{equation*}

where ${I}_{\mathrm{P}\left(\mathrm{u}\right)}$ and $\overline{I_{\mathrm{P}\left(\mathrm{s}\right)}}$ refer to soil fungal functional group indicators (the relative abundance of ECM fungi or pathogenic fungi) in each unsterilized soil from *P. elliottii* and the average indicators in four sterilized soils from *P. elliottii*. ${I}_{\mathrm{P}}$ or $\overline{I_{Cun}}$ refers to soil fungal community indicators, namely the relative abundance of ECM fungi or pathogenic fungi, in the live soil from *P. elliottii* and the average indicators in four live soils from *C. lanceolata*. Positive SFFs means that the relative abundance of ECM fungi or pathogenic fungi in the unsterilized soil from *P. elliottii* was higher than in the sterilized soil from *P. elliottii* or unsterilized soil from *C. lanceolata*, and vice versa.

### Statistical analysis

Alpha diversity was judged by community diversity using the Shannon-Weiner and Simpson diversity indices. Community richness was calculated using the Chao and Ace index (Chao et al. 2014). We also calculated the richness for two fungal functional group (ECM and pathogenic fungi). Beta diversity of soil fungal communities under different treatments was analyzed using Bray–Curtis distance and visualized using nonmetric multidimensional scaling (NMDS) (Clarke 1993). Bray–Curtis distance was also used in the principal coordinate analysis to analyze the composition of the community composition of two fungal functional group (ECM and pathogenic fungi). This analysis was conducted in R 4.1.0 using the ‘vegan’ and ‘ggplot2’ packages (R Core Team 2019). We finally performed indicator species analysis to determine these specific taxa in certain soil origins. It was conducted in R 4.1.0 using the *multipatt* function within the ‘indicspecis’ package (De Caceres and Legendre 2009).

Original and background data of soil abiotic properties and plant traits were analyzed by one-way ANOVA post hoc tests. Significance of PSF and SFF values was determined by fitting the linear regression model without intercept terms. This represented the differences in plant responses and fungal communities between unsterilized and sterilized conspecific soils or unsterilized conspecific and heterospecific soils. Linear regression model was conducted in R 4.1.0 using the *lm* function (R Core Team 2019). Differences between feedback values (RSAF and PRE) or SFF (the relative abundance of ECM fungi and pathogenic fungi) under the control and P addition were analyzed using independent-sample *t*-test. A two-tailed Pearson's correlation coefficient was used to test the respective relationship between SFFs in terms of two fungal functional groups and PSF on absorptions. To support the relationship between SFFs and PSF on absorptions, we also test the respective relationship between plant nutrient absorption (RSAF) and the richness or composition of two soil fungal functional groups. All statistical results were considered marginally significant at *P* < 0.1 and significant at *P* < 0.05. One-way ANOVA, *t*-test and Pearson's correlation coefficient were performed in SPSS 25.0 (IBM, Armonk, NY, USA). Figures were plotted using Origin 2021 (Origin Lab., Hampton, MA, USA).

## Results

### Absolute and relative PSFs

**Figure 1:**
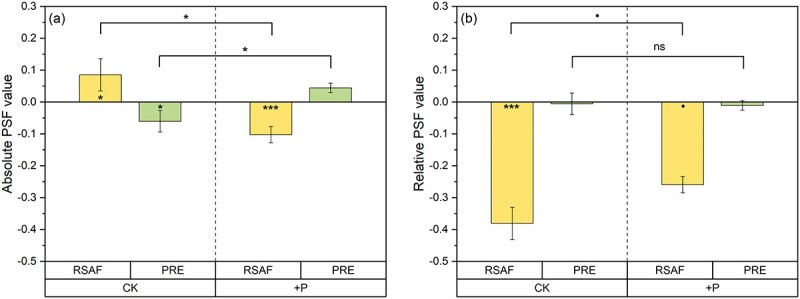
Absolute plant–soil feedbacks (PSFs) and relative PSFs (means ± SE) in terms of RSAF (yellow) and PRE (green). (a) The absolute PSFs refer to plant responses to unsterilized conspecific versus sterilized conspecific soil, and (b) the relative PSFs refer to plant responses to live conspecific versus live heterospecific soils in the control (CK) and phosphorus addition (+P). Significant feedbacks are displayed inside the column (Table S2 available as Supplementary data at *Tree Physiology* Online). Differences between the control and phosphorus addition are indicated by one-way ANOVA. ns, no significance, ·*P* < 0.1, ^*^P < 0.05 and ^*^^*^^*^P < 0.001.

Directions of absolute PSF on absorption and on resorption were significantly opposite (*P <* 0.05; Figure 1a). The absolute PSF on absorption was positive, whereas the effect on resorption was negative. However, both absolute PSFs were observed at relatively low magnitude. This meant that the trade-off between P absorption and P resorption was significant but weak. Phosphorus addition canceled the trade-off, and the absolute PSF on absorption became significantly negative (*P <* 0.001), whereas the absolute PSF on resorption was insignificantly positive. The absolute PSF on absorption was negative showing that P addition induced the increase of RSAF (8.4%) in the unsterilized conspecific soil compared with the dramatic increase (69.2%) of RSAF in the sterilized conspecific soil (*P <* 0.05; Table 1). Meanwhile, the insignificant absolute PSF on resorption means that there was a weak increase (4.8%) of PRE in the unsterilized soil and a weak decrease (17.0%) of PRE in the sterilized soil under P addition (Table 1).

**Table 1:** A summary of two phosphorus acquisition strategies (root–soil accumulation factor, RSAF and PRE), the relative abundance and the richness of two soil fungal guilds (ECM fungi and pathogenic fungi) for *P. elliottii* in three soil treatments (unsterilized conspecific, sterilized conspecific and unsterilized heterospecific soils) without (CK) and with phosphorus addition (+P).

Soil origin	Treatment	RSAF	PRE (%)	ECM fungi (%)	Pathogenic fungi (%)	ECM fungal richness	Pathogenic fungal richness
Unsterilized soil of *P. elliottii* (conspecific soil)	CK	3.58 (0.33)	28.28 (1.92)	21.79 (7.66)	0.87 (0.12)	8.00 (0.87)	70.00 (4.68)
	+P	3.88 (0.20)	29.64 (0.85)	20.29 (0.85)	2.53 (0.60)	10.00 (0.79)	65.75 (8.30)
Sterilized soil of *P. elliottii* (conspecific soil)	CK	2.89 (0.21)	32.20 (3.31)	18.40 (4.74)	2.81 (0.33)	6.25 (0.96)	62.25 (11.22)
	+P	4.89 (0.16)	26.73 (1.16)	2.73 (0.57)	1.28 (0.37)	6.25 (0.54)	55.50 (4.45)
Unsterilized soil of *C. lanceolata* (heterospecific soil)	CK	8.44 (0.73)	28.39 (3.87)	15.66 (5.29)	0.49 (0.15)	9.00 (0.35)	43.00 (1.84)
	+P	7.02 (0.92)	30.34 (5.12)	8.59 (0.61)	0.87 (0.17)	8.50 (1.15)	49.00 (1.06)

Values: mean (±SE)

The relative PSF on absorption was significantly negative (*P <* 0.001; Figure 1b). It represented that RSAF was much higher in the heterospecific soil than in the conspecific soil. However, the relative PSF on resorption was nearly neutral, exhibiting few differences in PRE between conspecific and heterospecific soil. Therefore, the relative PSF on absorption was stronger than the absolute PSF on absorption, and the relative PSF on resorption was weaker than the absolute PSF on resorption. Phosphorus addition only weakened the strength of the relative PSF on absorption but it was still marginally significantly negative (*P <* 0.1; Figure 1b). The RSAF was decreased by 16.8% in the heterospecific soil under P addition (*P <* 0.1; Table 1). Compared with the control, P addition had no effect on P resorption and slightly increased PRE by 6.9% in the heterospecific soil. Phosphorus addition did not change directions of relative absorption and PSF on resorptions.

### Absolute and relative SFFs

**Figure 2:**
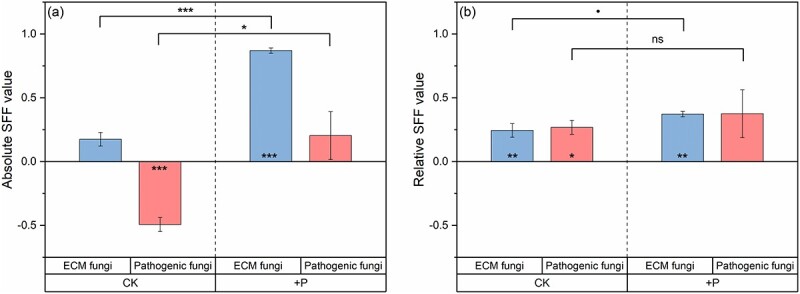
Absolute SFFs and relative SFFs (mean ± SE) in terms of the relative abundance of ECM fungi (blue) and pathogenic fungi (red). (a) The absolute SFFs refer to the responses of the fungal community to the unsterilized versus the sterilized soils conditioned by *P. elliottii* and (b) the relative SFFs refer to the responses of the fungal community to the live soil conditioned by *P. elliottii* versus the live soil conditioned by *C. lanceolata* in the control (CK) and phosphorus addition (+P). Significant feedbacks are displayed inside the column (Table S3 available as Supplementary data at *Tree Physiology* Online). Differences between the control and phosphorus addition are indicated by one-way ANOVA. ns, no significance, ·*P* < 0.1, ^*^P < 0.05, ^*^^*^P < 0.01 and ^*^^*^^*^P < 0.001.

Without P addition, the relative abundance of ECM fungi was 21.8 and 18.4% in the unsterilized and sterilized soil of *P. elliottii*, respectively (Table 1). Similar proportions of ECM fungi in two different soil treatments corresponded to the insignificant absolute SFF (Figure 2a). Phosphorus addition significantly strengthened the positive direction of absolute SFF (*P <* 0.001; Figure 2a). It referred to the significant difference between the relative abundance of ECM fungi in the unsterilized soil (20.3%) and sterilized (2.7%) soil from *P. elliottii* (Table 1). In the context of the relative abundance of pathogenic fungi, absolute SFF was negative (*P <* 0.001; Figure 2a). The relative abundance of pathogenic fungi was 0.9% in the unsterilized soil from *P. elliottii* and 2.8% in the sterilized soil from *P. elliottii* (Table 1). Compared with the control, the direction of absolute SFF for pathogenic fungi was shifted from negative to insignificantly positive under P addition (Figure 2a). The relative abundance of pathogenic fungi was low and similar in the unsterilized (2.5%) and sterilized (1.3%) soil.

In the unsterilized soil from *C. lanceolata*, the relative abundance of ECM fungi was 15.7%, and of pathogenic fungi was 0.5% (Table 1). Combined with the relative abundance of two fungal functional groups in the unsterilized soil from *P. elliottii*, the relative SFF was positive in terms of both ECM fungi (*P <* 0.01) and pathogenic fungi (*P <* 0.05; Figure 2b). The relative abundance of ECM fungi decreased to 8.6% in the soil from *C. lanceolata* under P addition (Table 1), and the relative SFF of ECM fungi enhanced the positive direction (*P <* 0.01; Figure 2b). Regarding pathogenic fungi, P addition slightly increased their relative abundance in the soil from *C. lanceolata* (0.9%; Table 1). The relative SFF in terms of pathogenic fungi became insignificantly positive, which meant no difference between the relative abundance of pathogenic fungi in the soil from *P. elliottii* and the soil from *C. lanceolata* under P addition (Figure 2b).

### Soil fungal community composition

Live soil from *P. elliottii* had the highest Shannon-Weiner index and the lowest Simpson index, followed by the sterilized soil from *P. elliottii* and the live soil from *C. lanceolata*. Phosphorus addition increased the Shannon-Weiner index and decreased the Simpson index in the live soil from *P. elliottii* (Figure S1 available as Supplementary data at *Tree Physiology* Online). However, the Shannon–Weiner index decreased and the Simpson index increased in the sterilized soil from *P. elliottii*. A high Chao and Ace index reading had no significant difference for the live soil from *P. elliottii* and *C. lanceolata*. However, the richness was slightly higher in the live soil from *C. lanceolata* than from *P. elliottii*. The sterilized soil from *P. elliottii* had the lowest richness. Phosphorus addition decreased community richness in the sterilized soil without affecting community richness in the two live soils.

Functional groups, which are indicative of fungal community composition, were pathogenic fungi in the unsterilized and sterilized soil from *P. elliottii*, and saprotrophic fungi in the unsterilized soil from *C. lanceolata*, which accounted for three in the top five taxa (Table S4 available as Supplementary data at *Tree Physiology* Online). The most frequent pathogenic indicator species is *Ophiocordyceps communis* especially in the unsterilized soil. *Mortierella hyalina* as the most indicative species in the saprotrophic fungi was exclusively not found in the unsterilized soil from *C. lanceolata*. The top four indicative species in the sterilized soil exhibited the characteristics of saprotrophic fungal guilds.

**Figure 3:**
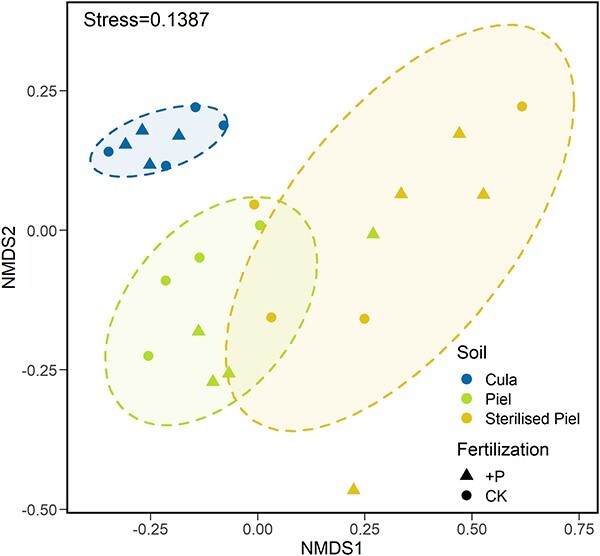
NMDS of fungal community in three soil treatments. Live heterospecific soil from *C. lanceolata* plantation (Cula, blue), live conspecific soil from *P. elliottii* plantation (Piel, green) and sterilized conspecific soil from *P. elliottii* plantation (sterilized Piel, yellow) under the control (CK, triangles) and phosphorus addition (+P, circles).

The soil fungal community in both the live and sterilized soils from the *P. elliottii* plantation was different from that from the *C. lanceolata* plantation (Figure 3). Community compositions were also different between soils from these two plantations. In the soil from the *P. elliottii* plantation, saprophytic fungi were the dominant functional group. However, more than half of the soil fungal community from the *C. lanceolata* plantation could not be defined (Figure S2 available as Supplementary data at *Tree Physiology* Online). In addition, sterilization did not alter the soil fungal community (Figure 3). The sterilized soil from *P. elliottii* had the most pathogenic fungi in the control among the three soil treatments (Figure S2 available as Supplementary data at *Tree Physiology* Online). The live soil from the *P. elliottii* plantation had the highest levels of ECM fungi, whereas other symbiotic fungi were the majority in the sterilized soil from the *P. elliottii* plantation, and the live soil from *C. lanceolata* plantation had the least. Phosphorus influenced the fungal functional composition, predominantly in the sterilized soil from *P. elliottii* plantation, leading to the presence of considerably more saprophytic fungi as well as far fewer ECM and pathogenic fungi.

### Correlations between P absorption and two soil fungal functional groups

**Figure 4:**
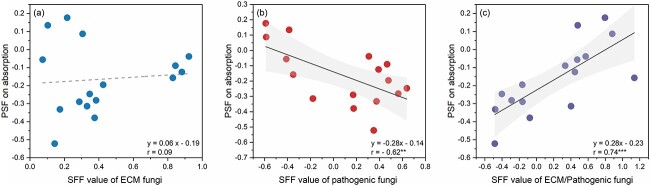
Relationships between two types of plant–soil feedbacks (PSFs) on absorption and SFF in terms of (a) the relative abundance of ECM fungi (blue dots), (b) pathogenic fungi (red dots) and (c) the relative abundance ratio of ECM fungi to pathogenic fungi (purple dots). Solid lines indicate linear regression, and shaded areas indicate 95% confidence intervals. The level of statistical significance: ^*^^*^P < 0.01 and ^*^^*^^*^P < 0.001.

In this study, we did not consider the impacts of P addition and different soil origins, but rather examined the role of ECM and pathogenic fungi in the P absorption of plants under the regulation of PSFs. Soil fungal factors of ECM fungi had an insignificant correlation with PSF on absorption. Soil fungal factors of pathogenic fungi had a significant negative correlation with PSF on absorption (*r* = −0.62, *P <* 0.01; Figure 4b; Table S5 available as Supplementary data at Tree Physiology Online). There was a positive correlation between PSF on absorption and SFF of ECM to pathogenic fungi (*r* = 0.74, *P <* 0.001; Figure 4c). The richness and composition of two fungal functional groups exhibited the insignificant relationship with RSAF, except the pathogenic fungal richness that had the negative relationship with RSAF (*r* = −0.37, *P <* 0.1; Figure S3 available as Supplementary data at *Tree Physiology* Online).

## Discussion

### Effects of PSF on P acquisition pathways

Disentangling plant nutrient acquisition pathways is central to understanding plant performance in the context of PSFs (Richardson et al. 2009, Bennett and Klironomos 2019). Our first hypothesis referred to positive absolute PSF on absorption, which is supported by the higher RSAF in the unsterilized soil compared with the sterilized soil (Figure 1a, Table 1). This may relate to interactions of microbes within the soil matrix, especially those rhizospheric fungi, such as mycorrhizal fungi, as well as pathogenic fungi (van der Putten et al. 2016). Ectomycorrhizal fungi can exchange surplus P for carbon from host plants. This process is particularly active in pine trees, leading to effective P absorption (Richardson et al. 2009, Zechmeister-Boltenstern et al. 2015, Hofmann et al. 2016). Therefore, plants tend to absorb P through mycorrhizal pathway in the unsterilized soil where ECM fungi thrive (Chuyong et al. 2000). This is evidenced by the higher RSAF in the unsterilized soil, which had a higher ECM fungal richness than in the sterilized soil (Table 1). In addition, ECM fungi directly suppress the activity of pathogenic fungi (Berendsen et al. 2012). Infection from pathogenic fungi can also be indirectly inhibited by ECM fungi through forming a special physical sheath and Hartig net (Bennett et al. 2017, Chen et al. 2019). This was demonstrated by the negative absolute SFF found in the pathogenic fungi, indicating that the relative abundance of pathogenic fungi was lower in the unsterilized soil in comparison with the sterilized soil (Figure 2a). The antagonism between ECM fungi and pathogenic fungi can be observed through the positive correlation between PSF on absorption and the relative abundance ratio of ECM fungi to pathogenic fungi (Figure 4c). Positive absolute SFF in ECM fungi and negative absolute SFF in pathogenic fungi supported higher P absorption in the unsterilized soil (Figure 2a).

Negative absolute PSF on resorption also confirmed our hypothesis that it took place in the opposite direction to positive absolute PSF on absorption (Figure 1a). Lower PRE corresponded with higher RSAF in the unsterilized soil compared with the sterilized soil (Table 1). This is in accordance with the theory that nutrient resorption and nutrient absorption are in a balance depending on the carbon cost invested by plants (Aerts and Chapin 2000, Wright and Westoby 2003). Plants can only provide a limited carbon source to drive P absorption by mutualistic fungi. Therefore, when P absorption requires too much carbon, the plants may rather acquire P through resorption, with the carbon saved being used for tissue growth and defense (Brant and Chen 2015, Kou et al. 2017).

Our second hypothesis was that specific pathogenic fungi in the conspecific soil led the relative PSF on absorption to become negative (Figure 1b). Lower RSAF in the unsterilized conspecific soil than in the unsterilized heterospecific soil supported the direction of relative PSF on absorption in the hypothesis (Table 1). The negative relative PSF on absorption may result from the increase of pathogenic fungi in long-term plantation monocultures rather than the presence of specific pathogenic fungi in the conspecific soil (Klironomos 2002, Bennett et al. 2012, van der Putten et al. 2016). Indicator species analysis in the two soil origins indicated no difference between the species of pathogenic fungi in conspecific and heterospecific soils (Table S4 available as Supplementary data at *Tree Physiology* Online). Other studies also demonstrated that root exudates from monoculture plants trigger the proliferation of native pathogenic fungi in conspecific soils (Li et al. 2010, Li et al. 2014). In addition, the infection capacity of pathogenic fungi constantly increases in the context of long-term monocultures (Nihorimbere et al. 2011). It is confirmed by higher relative abundance and higher richness of pathogenic fungi in the conspecific soil from *P. elliottii* plantation in comparison with the heterospecific soil from the *C. lanceolata* plantation (Figure 2b, Table 1). A significant build-up of pathogenic fungi in the conspecific soil caused root functional lesions, resulting in decreased P absorption (Raaijmakers et al. 2009, Lobmann et al. 2016). An alternative mechanism for the negative relative PSF on absorption could be the stimulation of proactive root absorption. It is due to non-cooperation in the heterospecific soil between plants and mycorrhizal fungi, which have monopolistic functions (Berendsen et al. 2012, Werner et al. 2014). The influence of species-specificity can be witnessed by the difference between fungal communities for the conspecific soil and the heterospecific soil, as shown in the results of the NMDS analysis (Figure 3). Therefore, P absorption by *P. elliottii* grown in the heterospecific soil from *C. lanceolata* plantation is more dependent on roots than on ECM fungi. The efficiency of root uptake is accelerated and promotes RSAF. Fewer pathogenic fungi from the heterospecific soil exhibited species-specificity because they had insufficient time to invade the alien host plant (Raaijmakers et al. 2009). The lower relative abundance of ECM fungi and pathogenic fungi in the heterospecific soil from *C. lanceolata* plantation supports our conjecture (Figure 2a).

No significant change in PRE was found for relative PSF in the control, which partly rejects our second hypothesis of the positive direction of relative PSF on resorption (Figure 1b). It indicates no difference between PRE in the conspecific soil and in the heterospecific soil (Figure 1b, Table 1). Given that soil microorganisms, especially soil fungi, induce PSF, the presence of insignificant relative PSF on resorptions suggests that the soil fungal community composition before being conditioned by two species could not explain the variation in P resorption. It demonstrates that the characteristics of the structure and function of soil microbiota are not associated with nutrient resorption (Li et al. 2016*b*).

### The trade-off between P acquisition pathways in PSF under P addition

Soil P availability regulates fungi-mediated PSFs and influences P acquisition (Castle et al. 2016, Gundale and Kardol 2021). Absolute PSF on absorption was altered from significantly positive to significantly negative due to P addition. It implies that P absorption was lower in the unsterilized soil than in the sterilized soil (Figure 1a, Table 1). There might be two reasons behind the variation of P absorption. First, sterilization would lead to the side effects of increased nutrient availability including available nitrogen (Pernilla Brinkman et al. 2010). Therefore, plants would invest more to P absorption in return, which finally results in the high P absorption in the sterilized soil. Second, higher P availability from P addition prompts plants to depend more on their roots and less on mycorrhizal fungi for nutrient uptake (Smith-Ramesh and Reynolds 2017, Zheng et al. 2017). Trades between mycorrhizal fungi and host plants are jointly controlled by the host plants and mycorrhizal fungi, following the rule of microbial markets (Werner et al. 2014). Therefore, the presence of more ECM fungi in the unsterilized soil immobilizes the excess P from exogenous P input and provides less P for the host plants until it becomes scarce again (Kiers et al. 2011, van’t Padje et al. 2020). However, roots in the sterilized soil can directly absorb the immobilized P from the ECM fungi. Even with a higher relative abundance and richness of ECM fungi, RSAF was still lower in the unsterilized soil compared with the sterilized soil (Table 1). The relative abundance and richness of pathogenic fungi were higher in the unsterilized soil compared with the sterilized soil (Figure S2 available as Supplementary data at *Tree Physiology* Online, Table 1). This provides an alternative explanation for the negative PSF on absorption. Furthermore, the traditional root diameter-based definition may underestimate the level of P absorption because fine roots (<2 mm) often comprise both transport and absorptive roots. This can be improved by adopting an order-based root definition where absorptive roots (first- and second-order) are more relevant in nutrient uptake (McCormack et al. 2015). In addition, the stronger negative feedback might arise from the 1-year-old seedling, because seedlings often suffer more from specialist pathogens than adult trees (Chen et al. 2019, Elger et al. 2009). Therefore, we should take into account either extending experimental duration or carrying out the PSF experiment on adult trees.

Phosphorus addition shifted absolute PSF on resorption from a strongly negative direction to a weakly positive one (Figure 1a). This is inconsistent with the strength hypothesis of this study that P addition would not change the direction of absolute PSF on resorption. The insignificant absolute PSF on resorption suggests that P addition weakens the trade-off between the two P acquisition pathways, as it leads to an increased PRE in the unsterilized soil and decreased PRE in the unsterilized soil (Table 1). This is consistent with two contrasting patterns between nutrient resorption and soil nutrient availability. Decreased PRE under P addition corresponded with the negative relationship of plant nutrient efficiency and soil nutrient availability (Tully et al. 2013, Yuan and Chen 2015, Ji et al. 2018). However, increased PRE is also in line with other studies that found a positive relationship (Nambiar and Fife 1987, Sabaté et al. 1995).

### Limited effects of P addition on P acquisition pathways in relative PSF

The relative PSF on absorption continued to show negative under P addition due to the absolute advantage of RSAF in the heterospecific soils (Figure 1b). This might result from the covariation of ECM fungi and pathogenic fungi in both conspecific and heterospecific soils (Figure S2 available as Supplementary data at *Tree Physiology* Online). Phosphorus addition would inhibit acid phosphatase activity, which often occurs in both plants and ECM fungi (Hofmann et al. 2016). This undermines the role of ECM fungi in P absorption. Alternatively, P addition decreases the level of mycorrhizal colonization because mutualistic symbiosis tends to be weak in nutrient-rich soils (Pernilla Brinkman et al. 2010, Zechmeister-Boltenstern et al. 2015, Mujica et al. 2020). Therefore, both lead to less carbon allocation from plants to mycorrhizal fungi and less dependence on them to capture P under P addition (Treseder 2004). This is demonstrated by the lower relative abundance of mycorrhizal fungi in both heterospecific and conspecific soils under P addition (Figure S2 available as Supplementary data at *Tree Physiology* Online). Reduction in ECM fungi induced the proliferation of pathogenic fungi owing to the loss of competition and antagonism directly or indirectly (in’t Zandt et al. 2019, Wei et al. 2018). Moreover, plants may invest more nutrients for growth than for defense when nutrient availability increases, making plants more vulnerable to pathogenic fungi (Endara and Coley 2011, Veresoglou et al. 2013, Heckman et al. 2016). This is supported by the increased relative abundance of pathogenic fungi in both conspecific and heterospecific soils under P addition (Figure S2 available as Supplementary data at *Tree Physiology* Online). Interestingly, we found P addition diminished the significance of relative PSF on absorption due to the decreased P absorption in the heterospecific or the increased P absorption in the conspecific soil (Figure 1b). The fungal community from the heterospecific soil responds rapidly to P addition, competes with plants for P and finally forces plants to reduce P uptake (Fan et al. 2020). However, the native soil fungal community can acclimate to the changing soil abiotic environment and cope with the P addition, leading to higher P absorption in the conspecific soil (Van Nuland et al. 2019).

In comparison with the control, no significant change occurred in relative PSF on resorption under P addition (Figure 1b). This resulted from the minor variation of RSAF in the trade-off between P resorption and P absorption (Table 1). A previous study demonstrated that soil microbial P increased considerably under P addition, indicating that P addition increased the demand of soil microorganisms for inorganic P (Turner and Joseph Wright 2013). More active microbial communities in the unsterilized soils compete intensely with plants for inorganic P, the only form available to the plants (Fujita et al. 2017). This pattern is rare in sterilized soil due to the inactive microbial community. In this case, appropriate extraneous inorganic P from P addition can be utilized by plants. As a result, the role of P addition in P absorption was limited, giving rise to little change in RSAF and PRE of plants grown in the unsterilized soil. Relative PSF on resorption under P addition is in line with the unchanged relative PSF on absorption. Owing to the fixed soil microbial P, P addition does not facilitate plant P absorption and has no effect on plant P resorption.

## Conclusions

Traditional research of PSF focuses more on plant growth (e.g., plant biomass) but overlooks plant nutrient acquisition (e.g., nutrient acquisition via absorption and resorption). Manipulative experiment of P addition was conducted to explore P acquisition pathways of plants under the framework of PSF. Plant–soil feedback affects aboveground P resorption and belowground P absorption through soil biotic and abiotic factors. Active P absorption acts as a medium for plants to perceive the external soil environments, influences the internal P resorption and finally contributes to the preference of P acquisition pathways. Soil sterilization forces *P. elliottii* to preferentially employ P resorption rather than P absorption in terms of the absolute PSF due to a relatively weak mycorrhizal symbiosis. Surprisingly, SFFs induce a strong trade-off between P absorption and P resorption in terms of the absolute PSF. *Pinus elliottii* grown in the heterospecific soil relies more on P absorption than P resorption according to the relative PSF. Pathogenic fungi play the most significant role in the conspecific soil fungal community that antagonize ECM fungi and underlie the decreased P absorption of plants. When soil abiotic factors are considered, P addition dilutes the effects of SFFs on the trade-off between two P acquisition pathways. However, P addition does not affect the trade-off in the relative PSF. Taken together, our findings facilitate a better understanding of the links between two P acquisition pathways in the context of PSF.

## Conflict of interest statement

The authors declare no competing interests.

## Funding

This work was supported by the National Natural Science Foundation of China [grant numbers 41830646]. The authors acknowledge the contributions of the anonymous reviewers.

## Authors’ contributions

N.M., L.K. and S.L. conceptualized and designed the experiment; N.M. performed the experiments with assistance from L.J., Y.X and J.Z.; N.M. conducted data analysis; N.M. and L.K. interpreted results; N.M., L.K. and S.L. wrote the original draft; X.D., S.M., X.F. and H.W. contributed revisions and edits, and all authors contributed to the writing of the manuscript.

## Supplementary Material

Supplementary_data_tpad044
