# Supplementary material for: Plant–soil feedback regulates the trade-off between phosphorus acquisition pathways in Pinus elliottii
Source: Tree Physiol. 2023 Apr 19;43(7):1092–103. doi: 10.1093/treephys/tpad044 (PMC10785040; doi:10.1093/treephys/tpad044)
Supplement: Supplementary_data_tpad044 [file supplementary_data_tpad044.docx]

Supplementary Data

**Plant-soil feedback regulates the trade-off between phosphorus acquisition pathways in *Pinus elliottii***

Ning Ma^a,b,c,†^, Liang Kou^b,d,†^, Shenggong Li^a,b,c,^*, Xiaoqin Dai^d^, Shengwang Meng^d^, Lei Jiang^a^, Jiajia Zheng^a,b^, Yafang Xue^a,b^, Xiaoli Fu^b,d^, Huimin Wang^b,d^

^a^ National Ecosystem Science Data Center, Key Laboratory of Ecosystem Network Observation and Modeling, Institute of Geographic Sciences and Natural Resources Research, Chinese Academy of Sciences, Beijing 100101, China

^b^ College of Resources and Environment, University of Chinese Academy of Sciences, Beijing 100049, China

^c^ Sino-Danish Center for Education and Research, Eastern Yanqihu Campus, University of Chinese Academy of Sciences, 380 Huaibeizhuang, 101400, Beijing, China

^d^ Qianyanzhou Ecological Research Station, Key Laboratory of Ecosystem Network Observation and Modeling, Institute of Geographic Sciences and Natural Resources Research, Chinese Academy of Sciences, Beijing 100101, China

*Authors for correspondence

†These authors contributed equally to this work

Tel.: +86 010 64889039; fax: +86 010 64868962; e-mail address: lisg@igsnrr.ac.cn

**Table S1** Physicochemical properties of three soil treatments after diluting with sand (Piel / Sterilized Piel: the unsterilized / sterilized soils from *P. elliottii* plantation; Cula: the unsterilized heterospecific soil from *C. lanceolata* plantation). Degrees of freedom (*df*) are also presented. **P* < 0.05, ** *P* < 0.01, and *** *P* < 0.001. Different letters among soil origin and nutrient addition treatments indicate significance based on one-way ANOVA post hoc tests. Significant *P-*values are presented in bold

| Soil treatments | pH | Available phosphorus  (mg/L) | NH_4_^+^-N  (mg/kg) | NO_3_^-^-N  (mg/kg) |
| --- | --- | --- | --- | --- |
| Piel  (Conspecific soil) | 5.85 (0.04)a | 0.88 (0.09) | 0.92 (0.07)b | 0.44 (0.04)a |
| Sterilised Piel  (Conspecific soil) | 5.66 (0.04)b | 0.90 (0.09) | 1.48 (0.09)a | 0.39 (0.03)ab |
| Cula  (Heterospecific soil) | 5.16 (0.01)c | 0.92 (0.07) | 0.70 (0.04)b | 0.23 (0.05)b |
| F (*df*=2) | **99.33***** | 0.01 | **24.36***** | **4.8*** |

**Table S2** Results of the two-factor linear model fitted without intercept terms to test for the significance of each absolute plant-soil feedback (absolute PSF) value and relative plant-soil feedback (relative PSF) value of each treatment combination. Significant P-values (*P* < 0.05) are presented in bold

|  |  | Absolute PSF | |  | Relative PSF | |  |
| --- | --- | --- | --- | --- | --- | --- | --- |
|  | *df* | t | *P* |  | t | *P* | |
| Control group ×PRE | 1 | -1.79 | **< 0.1** |  | -0.17 | 0.87 | |
| Control group ×RSAF | 1 | 2.52 | **< 0.05** |  | -11.28 | **< 0.001** | |
| Phosphorus addition group× PRE | 1 | 1.31 | 0.22 |  | -0.32 | 0.75 | |
| Phosphorus addition group× RSAF | 1 | -4.33 | **< 0.001** |  | 1.869 | **< 0.1** | |

**Table S3** Results of two-factor linear model fitted without intercept terms to test for the significance of each absolute soil fungal factor (absolute SFF) value and relative soil fungal factor (relative SFF) value of each treatment combination. Significant P-values (*P* < 0.05) are presented in bold

|  |  | Absolute SFF | |  | Relative SFF | |
| --- | --- | --- | --- | --- | --- | --- |
|  | *df* | t | *P* |  | t | *P* |
| Control group ×ECM fungi | 1 | -1.79 | 0.11 |  | 2.40 | **< 0.05** |
| Control group ×Pathogenic fungi | 1 | -4.84 | **< 0.001** |  | 2.63 | **< 0.05** |
| Phosphorus addition group× ECM fungi | 1 | 8.531 | **< 0.001** |  | 3.65 | **< 0.01** |
| Phosphorus addition group× Pathogenic fungi | 1 | 0.011 | 0.99 |  | -0.10 | 0.92 |

**Table S4** Indicator species analysis for soil fungal communities in the unsterilized (live) conspecific (Piel), sterilized conspecific (Sterilized Piel) soils from *P. elliottii* plantation and the unsterilized (live) heterospecific soil from *C. lanceolata* plantation (Cula). Fungal taxa that are significant indicators (*P* < 0.01) of community composition in each soil origin are shown (top five taxa)

| Soil origin | Indicator value | Taxonomy | Fungal Trophic guilds |
| --- | --- | --- | --- |
| Piel | 0.87 | *Ophiocordyceps communis* | Pathotroph |
|  | 0.81 | *Mortierella hyalina* | Saprotroph-Symbiotroph |
|  | 0.81 | *Ophiocordyceps communis* | Pathotroph |
|  | 0.81 | *Cladophialophora* | Saprotroph |
|  | 0.78 | *Ophiocordyceps communis* | Pathotroph |
| Sterilized Piel | 0.68 | *Mortierella hyalina* | Saprotroph-Symbiotroph |
|  | 0.64 | *Mortierella hyalina* | Saprotroph-Symbiotroph |
|  | 0.64 | *Teratosphaeriaceae* | Pathotroph-Saprotroph |
|  | 0.64 | *Exophiala xenobiotica* | Pathotroph-Saprotroph |
|  | 0.59 | *Ophiocordyceps communis* | Pathotroph |
| Cula | 0.92 | *Ophiocordyceps communis* | Pathotroph |
|  | 0.88 | *Botryobasidium* sp. | Saprotroph |
|  | 0.86 | *Ophiocordyceps communis* | Pathotroph |
|  | 0.81 | *Botryobasidium* sp. | Saprotroph |
|  | 0.78 | *Trichoderma guizhouense* | Saprotroph |

**Table S5** Primary data of soil abiotic properties and plant traits under the control group (CK) and phosphorus addition (+P). (Piel / Sterilized Piel: the unsterilized / sterilized soils from *P. elliottii* plantation; Cula: the unsterilized heterospecific soil from *C. lanceolata* plantation). Degrees of freedom (*df*) are also presented. **P* < 0.05, ** *P* < 0.01, and *** *P* < 0.001. Different letters among soil origin and nutrient addition treatments indicate significance based on one-way ANOVA post hoc tests. Significant *P-*values are presented in bold

| Soil origin | P addition levels | Soil nutrient concentration  (mg/g) | | | | |  | | Plant phosphorus concentration  (mg/g) | | | | |
| --- | --- | --- | --- | --- | --- | --- | --- | --- | --- | --- | --- | --- | --- |
|  |  | Total phosphorus | NH_4_-N | NO_3_-N |  | | | | Roots | | Senescent leaves | | Green leaves |
| Piel | CK | 0.13 b | 0.01 | 0.11 c | |  | | 0.46 (0.05) bc | | 0.37 (0.03) b | | 0.44 (0.02) c | |
|  | +P | 0.16 ab | 0.02 (0.02) | 0.11 (0.01) c | |  | | 0.62 (0.03) b | | 0.25 (0.02) ab | | 0.68 (0.44) ab | |
| Sterilized Piel | CK | 0.14 (0.01) ab | 0 | 0.17 (0.01) b | |  | | 0.41 (0.04) c | | 0.26 (0.04) b | | 0.53 (0.06) bc | |
|  | +P | 0.18 (0.01) a | 0.03 (0.01) | 0.13 c | |  | | 0.87 (0.09) a | | 0.41 (0.01) a | | 0.70 (0.03) ab | |
| Cula | CK | 0.06 c | 0.01 (0.01) | 0.15 (0.01) b | |  | | 0.46 (0.03) bc | | 0.36 (0.06) ab | | 0.63 (0.04) abc | |
|  | +P | 0.13 (0.03) b | 0.03 (0.02) | 0.17 (0.01) b | |  | | 0.82 (0.05) a | | 0.38 (0.06) ab | | 0.76 (0.09) a | |
| F | *df*=5 | **10.05***** | 0.73 | **14.53***** | |  | | **14.52***** | | **2.87*** | | **4.71**** | |

Values: Mean (±SE)


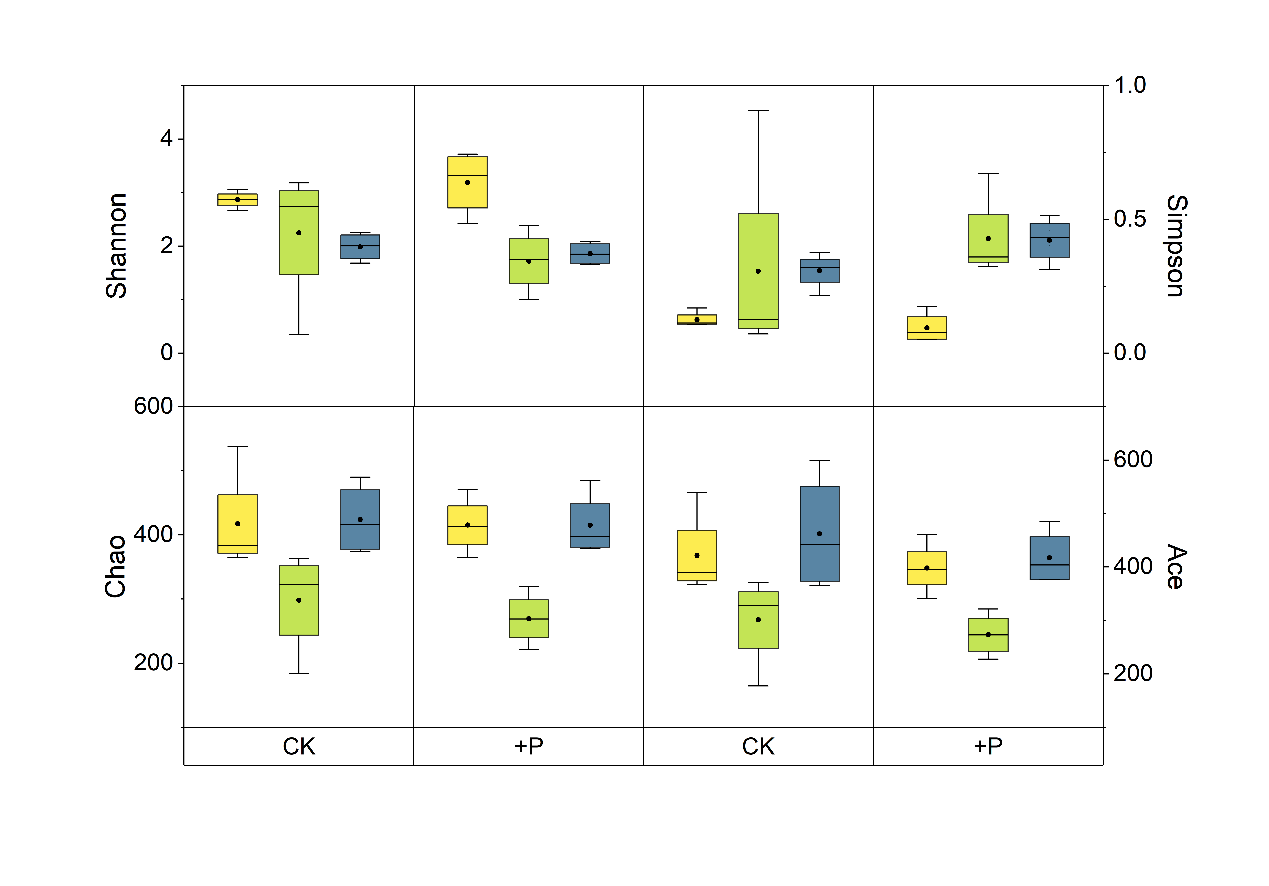


**Figure S1** Diversity of soil fungal community diversity: Shannon index and Simpson index (upper panels), and Chao richness and Ace richness (lower panels) in the unsterilized (live) conspecific (yellow), sterilized conspecific (green) soils from *P. elliottii* plantation and the unsterilized (live) heterospecific soil from *C. lanceolata* plantation (blue) without (CK) and with phosphorus addition (+P). Solid dots represent mean values. Boxplots show the minimum (lowest whisker), first quartile, median, third quartile and the maximum (upper whisker)


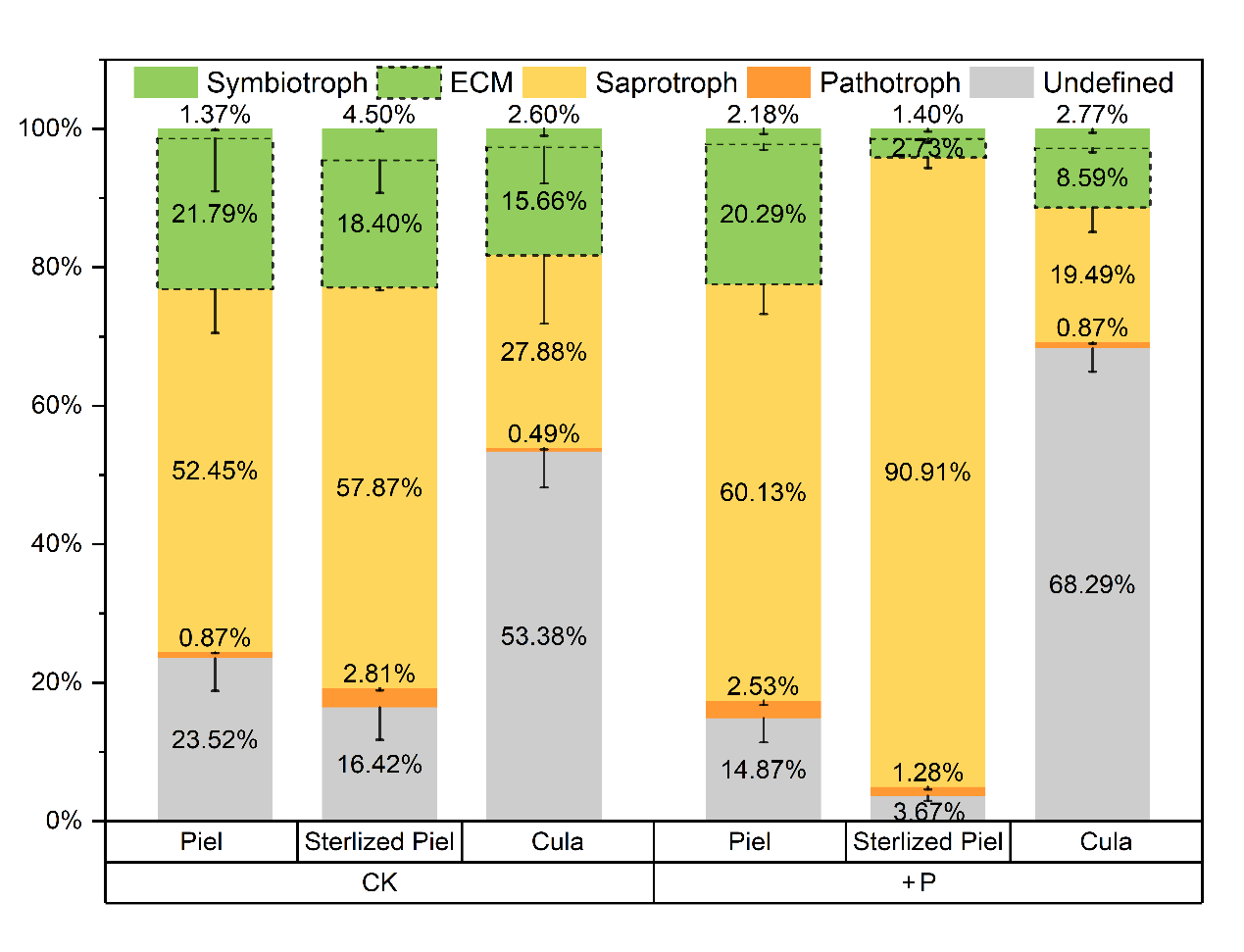


**Figure S2** Relative abundance of fungal trophic guilds: symbiotroph (green, ECM fungi within the dotted lines), saprotroph (yellow), pathotroph (orange), and the undefined (grey) in the unsterilized (live) conspecific (Piel), sterilized conspecific (Sterilized Piel) soils from *P. elliottii* plantation and the unsterilized (live) heterospecific soil from *C. lanceolata* plantation (Cula) without (CK) and with phosphorus addition (+P). The percentage represents the proportion of each trophic guild. All data are presented as the mean-SE


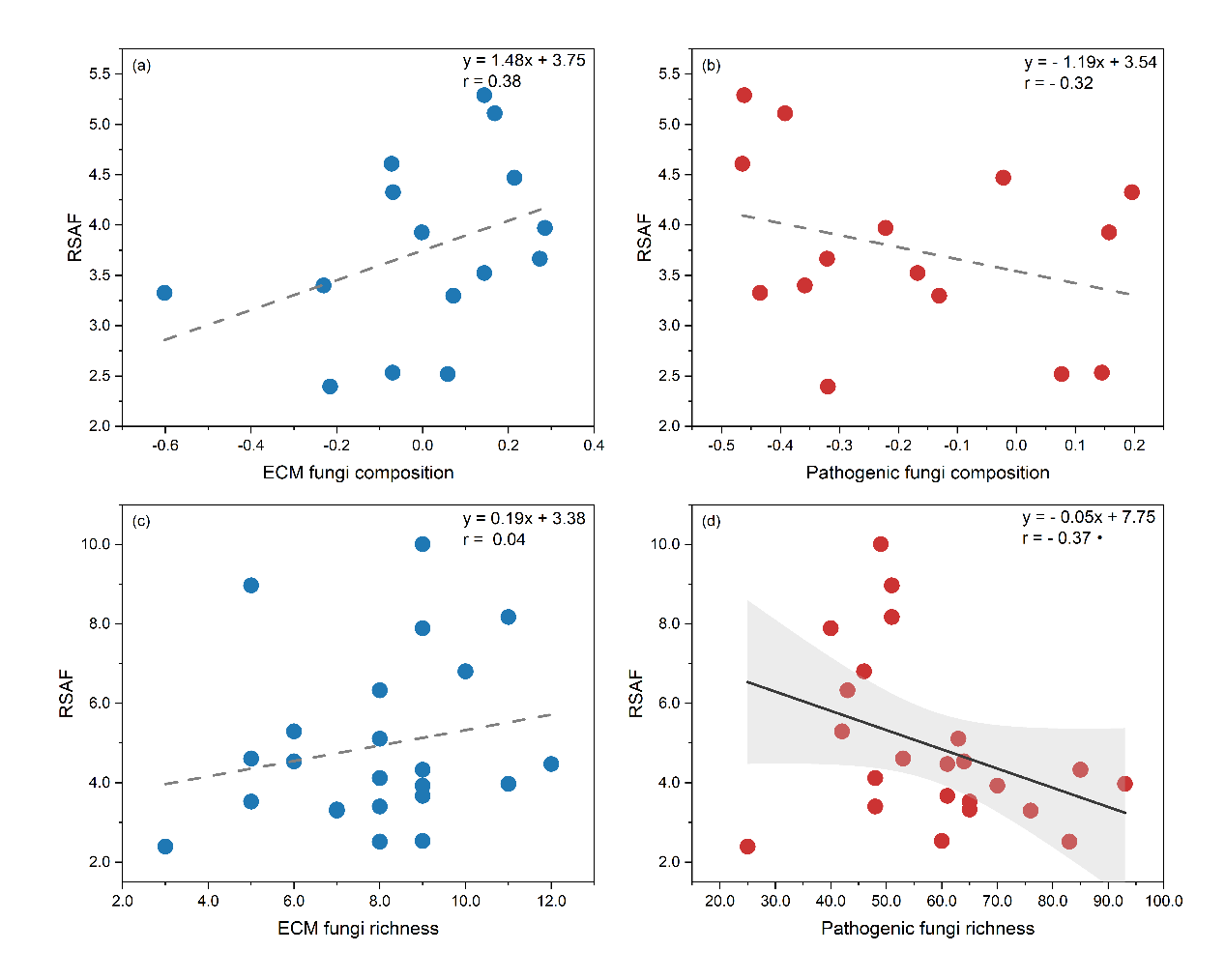


**Figure S3** Relationships between soil fungal community (ECM and pathogenic fungi) and root-soil accumulation factor (RSAF). (a-d) ECM or pathogenic fungi composition refers to the first principal coordinate of ECM or pathogenic fungi communities. · *P* < 0.1
